# Immiscible Viscous Fingering: The Simulation of Tertiary Polymer Displacements of Viscous Oils in 2D Slab Floods

**DOI:** 10.3390/polym14194159

**Published:** 2022-10-04

**Authors:** Alan Beteta, Ken S. Sorbie, Arne Skauge

**Affiliations:** 1 Institute of GeoEnergy Engineering, Heriot-Watt University, Edinburgh EH14 4AS, UK; 2 Energy Research Norway, 5007 Bergen, Norway

**Keywords:** viscous fingering, immiscible displacement, polymer flooding, enhanced oil recovery

## Abstract

Immiscible viscous fingering in porous media occurs when a high viscosity fluid is displaced by an immiscible low viscosity fluid. This paper extends a recent development in the modelling of immiscible viscous fingering to directly simulate experimental floods where the viscosity of the aqueous displacing fluid was increased (by the addition of aqueous polymer) after a period of low viscosity water injection. This is referred to as tertiary polymer flooding, and the objective of this process is to increase the displacement of oil from the system. Experimental results from the literature showed the very surprising observation that the tertiary injection of a modest polymer viscosity could give astonishingly high incremental oil recoveries (IR) of ≥100% even for viscous oils of 7000 mPa.s. This work seeks to both explain and *predict* these results using recent modelling developments. For the 4 cases (µ_o_/µ_w_ of 474 to 7000) simulated in this paper, finger patterns are in line with those observed using X-ray imaging of the sandstone slab floods. In particular, the formation of an oil bank on tertiary polymer injection is very well reproduced and the incremental oil response and water cut drops induced by the polymer are very well predicted. The simulations strongly support our earlier claim that this increase in incremental oil displacement cannot be explained solely by a viscous “extended Buckley-Leverett” (BL) linear displacement effect; referred to in the literature simply as “mobility control”. This large response is the combination of this effect (BL) along with a viscous crossflow (VX) mechanism, with the latter VX effect being the major contributor to the recovery mechanism.

## 1. Introduction

Viscous fingering, which is often referred to as the Saffman-Taylor instability [1], occurs when a viscous fluid is displaced by a less viscous fluid (either miscible or immiscibly). In this work, we focus on immiscible viscous fingering since this has proved to be the most difficult to simulate numerically using continuum numerical simulation methods. The understanding of when viscous instabilities occur and their impact on displacement is of critical importance to a range of applications, e.g., oil recovery [2], CO_2_ storage [3], hydrogen storage [4], and natural & engineered mixing process [5], amongst many others. Of particular interest in this work is immiscible oil/water displacements in porous media, where the initial unfavorable viscosity ratio (µ_o_/µ_w_) is reduced after a given time period to a somewhat more stable ratio (but *not* a stable ratio). This is precisely the process which occurs in tertiary polymer flooding of viscous oils.

Due to its complexity, the modelling of unstable displacement has received considerable attention in the literature for both miscible and immiscible processes. A range of analytical [6,7], numerical [8,9,10] and pore scale [11,12] approaches have been taken to capture the dynamics of unstable displacement. The classical review of viscous fingering by Homsy (1987) [13] has recently been updated by Pinilla et al. (2021) [14]. Additionally, a recent review of viscous fingering relating more closely to the present work is given in Salmo et al., (2022) [15]. This review shows that many of the methodologies put forward in the literature are either unable to reproduce *all* aspects of the fingering process at a range of length scales (i.e., oil/water production, pressure drop and finger patterns) or are not compared directly against experiments for full validation. Sorbie et al. (2020) [16] proposed a methodology to capture the main features of unstable, immiscible displacements using conventional numerical simulation software. The key steps are as follows:

- Define a fractional flow (f_w_*) with a shock front saturation (S_wf_) in line with the water saturation observed in the fingers, this fractional flow defines a unique ratio, (k_rw_/k_ro_), of relative permeabilities (RPs);
- Choose the actual RPs by maximising the total mobility function of the system (λ_T_(S_w_) = (k_rw_(S_w_)/ µ_w_) + (k_ro_(S_w_)/ µ_o_));
- Utilise a random correlated permeability field with a defined correlation length and permeability distribution such that the properties of the porous media are well represented;
- Perform 2D (or 3D) simulations in a sufficiently fine grid such that Δx and Δy are smaller than the correlation length defined above.

At this point it is important to contrast the observations and rationale from Sorbie et al. (2020) [16] against those put forward previously in the literature. Firstly, that relative permeabilities for viscous oils are typically measured using unsteady state experiments that are unlikely to generate the “true” relative permeability due to the unstable nature of the displacement [17]. Maini (1998) [17] goes on to show the significant effect flow rate can have on the determined relative permeabilities even at “low” viscosity ratios (µ_o_/µ_w_ = 42). Secondly, the results of these unsteady state experiments are typically input into simple 1D simulations to generate the relative permeability [18] or Buckley-Leverett analysis used for adverse viscosity ratios e.g., Blunt (2016) [19] examines the water saturation profile for viscosity ratios of up to 200, where it is likely there is significant instability. The approach proposed by Sorbie et al. (2020) [16], seeks to correct some of these issues by generating a fractional flow representative of the system and determining a set of relative permeabilities that give that fractional flow with a *high* total mobility (i.e., generates a path of least resistance for the water flood). Previous authors have observed the relationship between relative permeability, fractional flow, total mobility and the degree of instability [20], but have not connected these elements to a detailed methodology or compared against experimentally observed viscous fingers.

In order to reduce the impact of viscous fingering in oil recovery operations, it is possible to viscosify the injection water with polymers [21,22], such as hydrolysed polyacrylamide (HPAM), xanthan, scleroglucan [23]. Typically, the improvements to recovery are attributed to “mobility control”, however recent research has suggested that a viscous crossflow mechanism (VX) may be contributing significantly to the polymer improved recovery of viscous oils [16,24].

In the current paper the new simulation methodology presented by Sorbie et al. (2020) [16] is applied to a series of unstable immiscible displacement experiments from the literature [25,26] where a viscous oil (µ_o_ = 412 mPa.s–7000 mPa.s) is displaced first by water (µ_w_ = 0.9 mPa.s–1.0 mPa.s) then by a water phase viscosified by the addition of a hydrolysed polyacrylamide, HPAM (µ_p_ = 18 mPa.s–60 mPa.s). A previous paper applied our approach to the viscous unstable waterfloods in these experiments [15], and here this work is extended to the modelling of the tertiary polymer flooding (i.e., polymer injection following some period of water injection). Using this approach, we show in this paper that all stages of these experiments are captured (oil recovery, water cut, and pressure drop vs. PV injected), including representative finger patterns. These results were generated using a standard commercial numerical simulator (CMG STARS v2021.1) [27]. This represents a significant advance in terms of the simulation of unstable immiscible displacement, as these particular experiments have received much attention in the literature and other attempts have been made to directly match the oil recovery, water cut, pressure drop and finger patterns with mixed levels of success [28,29,30].

## 2. Materials and Methods

The experimental methodology and results have previously been described in detail in the literature [25,26]. However, a brief description is provided here for clarity. Slabs of Bentheimer sandstone (~30 cm × 30 cm for all cases other than 412 mPa.s which used ~15 cm × 15 cm) were saturated with a range of viscous crude oils (412 mPa.s–7000 mPa.s), aged to condition the rock towards oil wettability, before the oil was displaced by water flood and a subsequent tertiary polymer flood. Visualisation of the in situ saturations was performed using X-ray scanning throughout all stages of displacement.

In this work, those experiments are directly recreated in the simulator (CMG STARS) and the oil production, water cut, pressure drop and finger patterns matched to the experimental observations using the methodology summarised briefly above [16].

**Figure 1:**
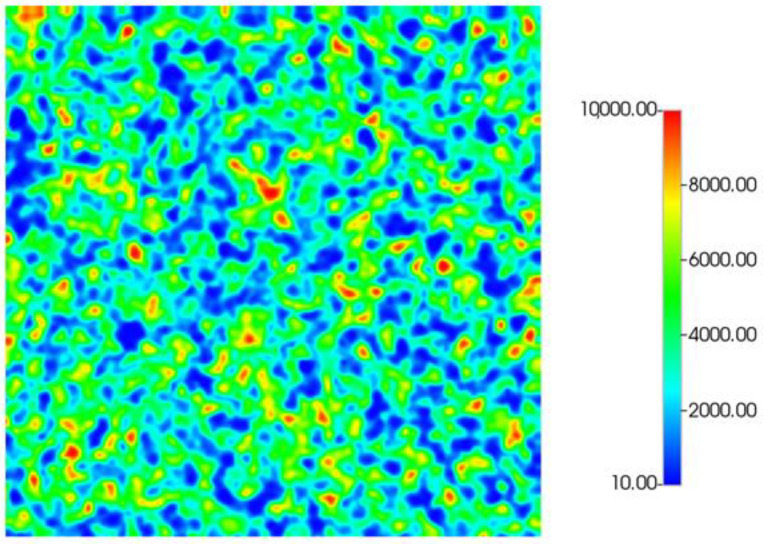
Base case permeability field—500 × 500 cells, $\lambda_{D}$ = 0.03.

In this work, the 2D simulation grid is generated with a dimensionless correlation length ($\lambda_{D}$) of 0.03 with 500 × 500 grid cells. This was established in previous work for the water displacements [15]. The base case simulation permeability field is shown in Figure 1. Permeability distribution ranges from 0.01 to 10 D with an average of ~3 D. The effect of this permeability range of (k_max_/k_min_) is examined below. Sensitivities to grid resolution and correlation length have been previously presented, and as such are not repeated here [15,16,31].

Relative permeability curves are generated using the LET correlations [32] to give greater flexibility in the shape of the curves. The form of the water ($k_{rw})$ and oil ($k_{ro})$ relative permeability curves are given in Equation (1).

(1) $$k_{rw}=k_{rw}^{0}\frac{S_{wn}{}^{L_{w}^{o}}}{S_{wn}{}^{L_{w}^{o}}+E_{w}^{o}{\left(1-S_{wn}\right)}^{T_{w}^{o}}}\;\mathrm{and}\;k_{ro}=k_{ro}^{0}\frac{{\left(1-S_{wn}\right)}^{L_{o}^{w}}}{{\left(1-S_{wn}\right)}^{L_{o}^{w}}+E_{o}^{w}S_{wn}{}^{T_{o}^{w}}}$$

where $k_{rw}^{0}$ and $k_{ro}^{0}$ are the water and oil relative permeability end points, respectively; L, E and T are constants; and $S_{wn}$ is the normalised water saturation ($S_{w}$) in Equation (2).

(2) $$S_{wn}=\frac{S_{w}-S_{wi}}{1-S_{wi}-S_{or}}$$

where $S_{wi}$ is the irreducible water saturation and $S_{or}$ is the residual oil saturation.

The fractional flow of water ($f_{w}\left(S_{w}\right)$) is given by the two forms shown in Equation (3).

(3) $$f_{w}\left(S_{w}\right)=\frac{1}{1+\left(\frac{k_{ro}.\mu_{w}}{k_{rw}.\mu_{o}}\right)}=\frac{M\left(S_{w}\right)}{M\left(S_{w}\right)+1}$$

where $M\left(S_{w}\right)$ is the mobility ratio at a given water saturation, Equation (4).

(4) $$M\left(S_{w}\right)=\left(\frac{\lambda_{w}}{\lambda_{o}}\right)=\left(\frac{k_{rw}.\mu_{o}}{k_{ro}.\mu_{w}}\right)\;$$

where λ_i_ the mobility of the phase specified by the subscript i = o or w for oil or water, respectively. The mobility ratio at the shock front saturation, S_wf_, denoted $M\left(S_{wf}\right)$ is of particular interest.

It is useful to revisit the topic of relative permeability curves at this point, and why the relative permeability curves in this work, and preceding work following the same methodology, have taken non-traditional forms. In order to generate fractional flow curves with appropriate water saturations and mobilities, along with maximising the total mobility, it is necessary to have more flexibility in the form of the relative permeability curves. As an example, when using the Corey-Brooks form of relative permeability, with only 1 exponent, it was not possible to generate a fractional flow representative of the observed finger patterns in the 412 mPa.s displacement case (finger water saturation ~0.4) while still matching the oil recovery, water cut, and pressure drop. Rather, those parameters were only able to be matched using a relative permeability set that resulted in a low water saturation in the fingers—leading to the wispy fingers discussed by Sorbie et al. (2020) [16]. Conversely, for the 2000 mPa.s case it was possible to achieve a match to all elements of the experiment using conventional relative permeability curve. This is likely due to the low water saturation present in the fingers (~0.2) in this high viscosity ratio case—such a fractional flow can be easily achieved with Corey-Brooks relative permeability curves. This highlights the requirement for a detailed understanding of the fractional flow of an unstable system to correctly simulate the displacement process and further study given to the role and form of the relative permeability curves, beyond the scope of this publication. Unfortunately, there are few representative examples in the literature where cm–m scale displacement experiments are carried out in true porous media with oil recovery, water cut, pressure drop and in situ imaging reported. As such, there is a limitation on how much of this investigation can be performed at the current date.

The viscosities of oil, water and polymer solutions in the 2D immiscible slab flood experiments are given in Table 1.

As opposed to the complex in situ rheology observed in porous media for HPAM, where both shear-thinning and shear-thickening are observed [33,34], the polymer solution is treated as a Newtonian fluid (i.e., not shear thinning or thickening). Given the low frontal advance rate in these experiments ~0.005 m/day, it is expected that there will not be sufficient variance in shear rate to significantly impact the in situ viscosity. A low sensitivity to injected viscosity was noted by Beteta et al. (2022) [31] in similar permeability Bentheimer cores during displacement of oil and direct simulation matches of the experiments were obtained using the methodology from Sorbie et al. (2020) [16] assuming a Newtonian fluid.

It should be noted that Vik et al. (2018) [35] observed a large difference in finger pattern and oil recovery during direct injection (i.e., at S_wi_ - secondary polymer flooding) with a range of fluids with varied in situ rheology: HPAM (shear-thinning and shear-thickening); low molecular weight HPAM (near-Newtonian); xanthan (shear-thinning); glycerol (Newtonian); water (Newtonian). It was shown that the highly visco-elastic HPAM showed the most stable displacement front and highest recovery, however as it has been possible to reproduce all experimental observations in the presented simulations it is not clear how this behaviour translates to *tertiary* polymer flooding. Although the results presented by Vik et al. (2018) [35] have been matched using pore network models previously in the literature [36]. However, the role of the polymer in situ rheology is a key area for future study.

A constant polymer adsorption of 10 µg/g has been applied to each simulation case. There is little sensitivity to the adsorption level for this range of values. The initial adsorption of 10 µg/g was therefore applied, as such this value has been selected based upon available literature data [23,37,38]. Similarly, a residual resistance factor (RRF) was only recorded for 1 case (2000 mPa.s) of 4.9. This RRF is applied to the 2000 and 7000 mPa.s cases, while a lower value of 2.5 is applied to the 412 and 616 mPa.s cases.

Experimental results will be presented later in this paper, but before doing so, we present the summary oil recovery data for the water and polymer flood displacements in Table 2. Note that since the full waterflood to several PV of injection were not performed in the experiments, the final waterflood recoveries are estimated (denoted EW) but the tertiary polymer flood recoveries (PR) were taken as the measured values. We define the incremental recovery (IR as a %) due to polymer displacement as:

IR = 100 × (PR − EW)/EW(5)

Of particular note for these displacements is that IR ≥ 100% for each case in Table 2. These IR values are extremely high for *any* EOR process, and these numbers are certainly target values for our modelling predictions presented below. These IR responses appear to increase as the oil viscosity increases but broadly increasing polymer viscosities were also used for the higher oil viscosities as shown in Table 2.

In all cases presented here, capillary pressure has been excluded from the modelling calculations as the system is presumed to be viscous dominated. While capillarity is known to stabilise immiscible displacement at short length scales [39,40], the in situ imaging acquired during the experiments upon which these simulations are based, shows clear viscous fingering. The authors are currently preparing a publication which will examine the role of capillary pressure and wettability on some of the cases presented here.

## 3. Results

### 3.1. Sensitivity to Permeability Range

Before presenting the detailed simulations of the 2D experiments, a number of sensitivity calculations are performed to demonstrate the rationale for the permeability range used in this study. These examples all use the input parameters derived for the 2000 mPa.s case, which is presented in detail later in this paper.

The permeability field is numerically re-scaled to a range of k_min_–k_max_ values using Equation (6) such that the permeability range moves from the base case of 1:1000 to 1:2. Rescaling the permeability field in such a way maintains the exact permeability field *structure* through all sensitivity calculations. The k_min_ and k_max_ values used have been selected to maintain an average permeability of ~3 D.

(6) $$k_{i2}=k_{min.2}+\left(\frac{k_{i1}-k_{min.1}}{k_{max.1}-k_{min.1}}\right)\left(k_{max.2}-k_{min.2}\right)$$

**Figure 2:**
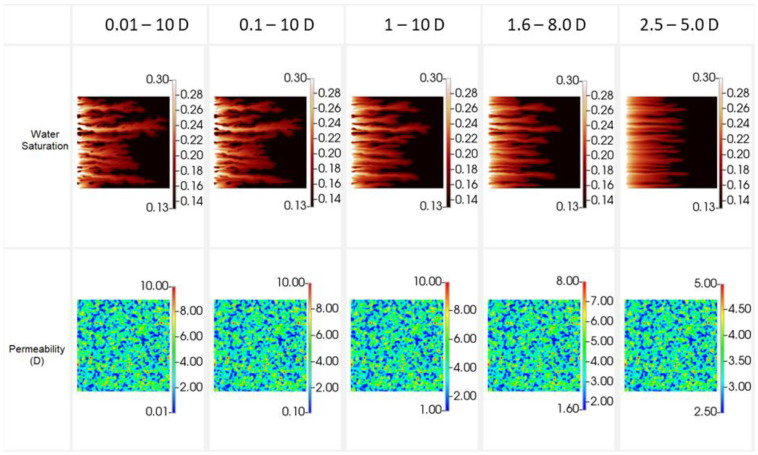
Sensitivity to permeability range for displacement of 2000 mPa.s oil—water saturation (**top**) and permeability (**bottom**).

**Figure 3:**
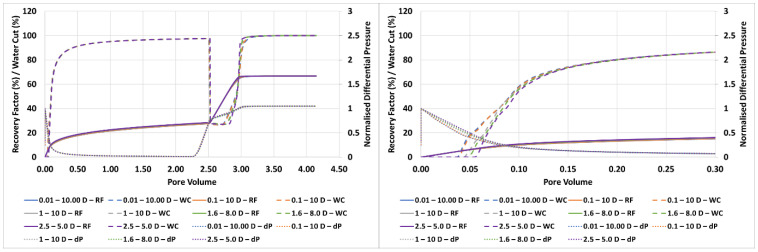
Oil recovery, water cut, and pressure drop during displacement of 2000 mPa.s oil at a range of permeability distributions. Full water and polymer flood (**left**) and early breakthrough (**right**).

The resulting finger patterns prior to water breakthrough (~0.04 PV) are shown in Figure 2 along with the respective permeability field to reinforce that the changes in finger patterns are solely due to the permeability values themselves. Similarly, Figure 3 shows oil recovery, water cut, and pressure drop for each of the cases.

It is clear from Figure 2 that the permeability range utilised in the simulation grid can have a significant impact once the ratio of k_min_:k_max_ is less than 1:5. A lower ratios the finger patterns begin to become more linear and by a ratio 1:2 show very little of the dendritic behaviour observed during highly unfavourable displacement. However, on examination of Figure 3 it is clear that while k_min_:k_max_ affects the finger patterns, little impact is observed on the oil recovery, water production and pressure drop. Of course, as the fingers blunt the front is more stabilised and the water breakthrough is slightly delayed at low k_min_:k_max_ ratios. This in fact simplifies the simulation process when simulating the unstable immiscible displacement processes detailed here—a precise understanding of the *exact* permeability distribution is not required, but rather through observation of the finger patterns the permeability range can be deduced., i.e., the correct permeability distribution is the one that gives representative finger patterns. Indeed, it is possible to match the production data and pressure drops with a wide range of permeability values if in situ imaging is not available as a constraint.

As a result of this sensitivity calculation and those previously performed in the literature, a 500 × 500 grid, λ_D_ = 0.03 and k_min_–k_max_ = 1:1000 have been used throughout as it provides the most representative finger patterns when compared to the experiments.

### 3.2. Displacement of the µ_o_ = 2000 mPa.s Oil

**Figure 4:**
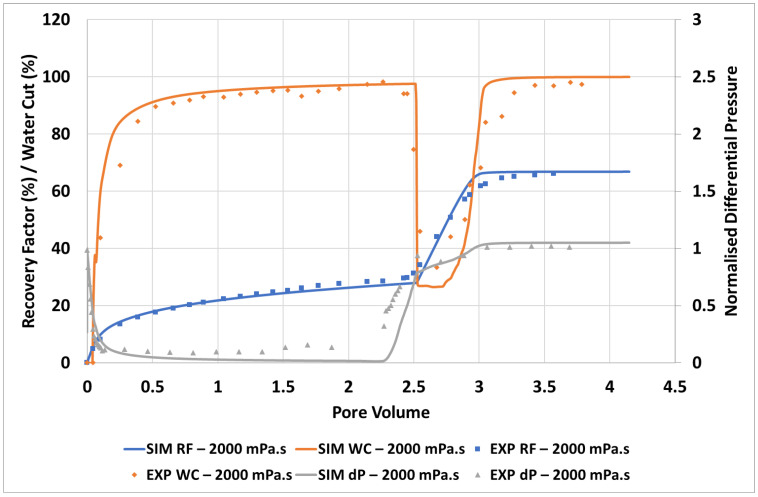
Simulation match of the unstable displacement of 2000 mPa.s oil.

The simulation match to the experimental data for the 2000 mPa.s case is given in Figure 4.

It can be seen from Figure 4 that the approach used here can be utilised to obtain an excellent match to the experimental oil recovery, water cut, and differential pressure observed in the experiment. It should be noted that there is some mismatch in differential pressure at the end of the water flood (~1.5–2.25 PV) however this remains in the limits of experimental error. Importantly, both the timing and magnitude of polymer response are very well captured here. The large, quick response to the injection of a moderate viscosity polymer solution (µ_p_ = 60 mPa.s) is in line with the viscous crossflow mechanism proposed by Sorbie and Skauge (2019) [24], as the displacement is still operating a strongly unfavourable viscosity ratio, (µ_o_/µ_p_) = 2000/60 ~ 333, although there is a bank of connate and injected water in front of the polymer bank.

**Figure 5:**
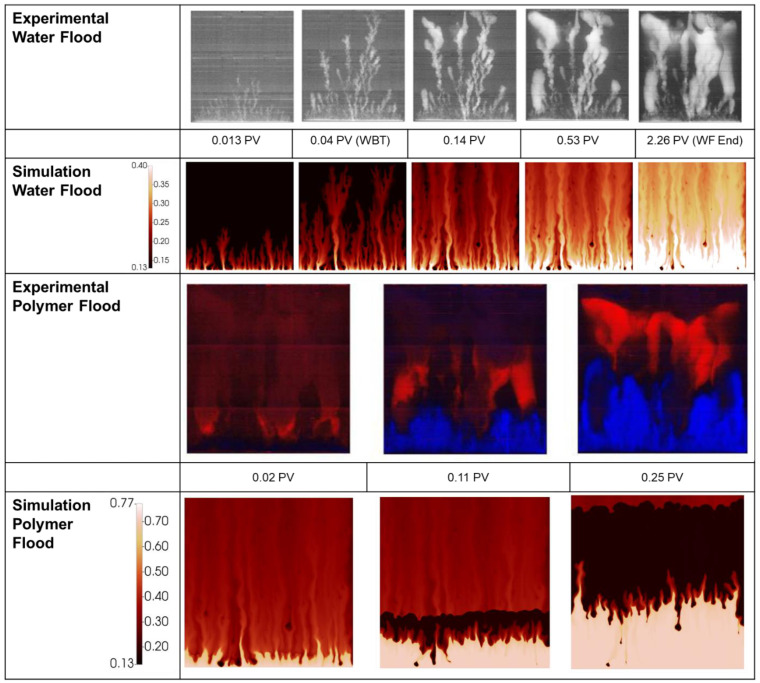
Experimental vs. simulated finger patterns during displacement of 2000 mPa.s oil.

The simulated finger patterns are compared with experimental observations in Figure 5. During the polymer flood, the experimental images are produced by subtraction of a reference image of the end of the water flood (image at 2.26 PV in Figure 5)—blue indicates an increase in water saturation whereas red indicates an increase in oil saturation.

There is good agreement between the simulated finger patterns during the early stages of the water flood in that approximately the correct number of fingers are observed and the dominant fingers show both spreading and tip splitting. At later stages (>0.14 PV) the simulated fingers are slightly more disperse than the experimental images. It may be a limitation of the simulator/methodology not able to catch the collapse of fingers into channels of high-water saturation. These channels formed are important for initiation of the viscous crossflow mechanism and the resultant acceleration of oil production by polymers. However, although they are not perfectly resolved in the late time finger images, they do result in the correct amounts of incremental oil (IR) recovered by the polymer in this case (see Figure 4) and in all later cases.

As the simulation moves to displacement by the polymer viscosified water phase, the simulation provides a good approximation of the saturations. At 0.11 PV there is a somewhat reduced oil bank vs. the experimental image, however by 0.25 PV the simulation is in line with the experiment distributions. The simulation captures the building of an oil bank along with the unstable front of the aqueous polymer phase (which is clearly still not completely stable).

**Figure 6:**
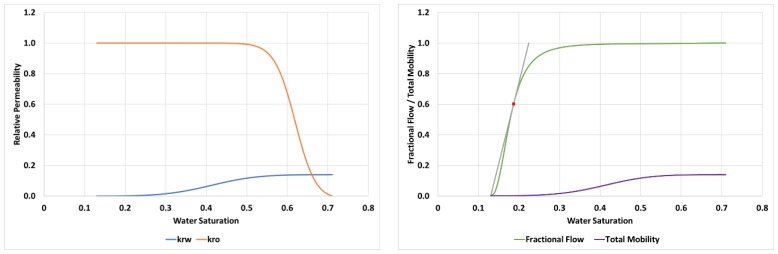
Relative permeabilities (**left**) and fractional flow & total mobility (**right**) for the displacement of 2000 mPa.s oil.

The input parameters required to achieve this match for the 2000 mPa.s oil case are detailed in Table 3 and the resultant relative permeabilities, fractional flow and total mobility functions are shown in Figure 6.

From the fractional flow in Figure 6 the water saturation at the shock front of the finger ($S_{wf}$) can be calculated from the tangent as, *S_wf_* = 0.19 which has a corresponding value of $M\left(S_{wf}\right)$ = 1.5.

### 3.3. Displacement of the µ_o_ = 7000 mPa.s Oil

**Figure 7:**
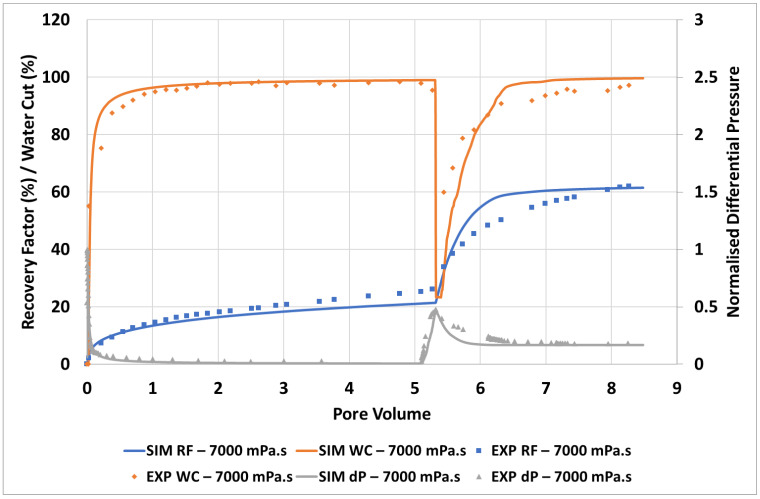
Simulation match of the unstable displacement of 7000 mPa.s oil.

Figure 7 shows the simulation match for the 7000 mPa.s oil viscosity case.

As with the 2000 mPa.s case, very good agreement is observed between simulation and experiment for recovery factor, water cut, and pressure drop. The extremely strong response to a modest increase in displacement fluid viscosity (1 to 60 mPa.s) relative to the defending fluid viscosity (7000 mPa.s) is well captured and supports the proposed viscous crossflow mechanism.

**Figure 8:**
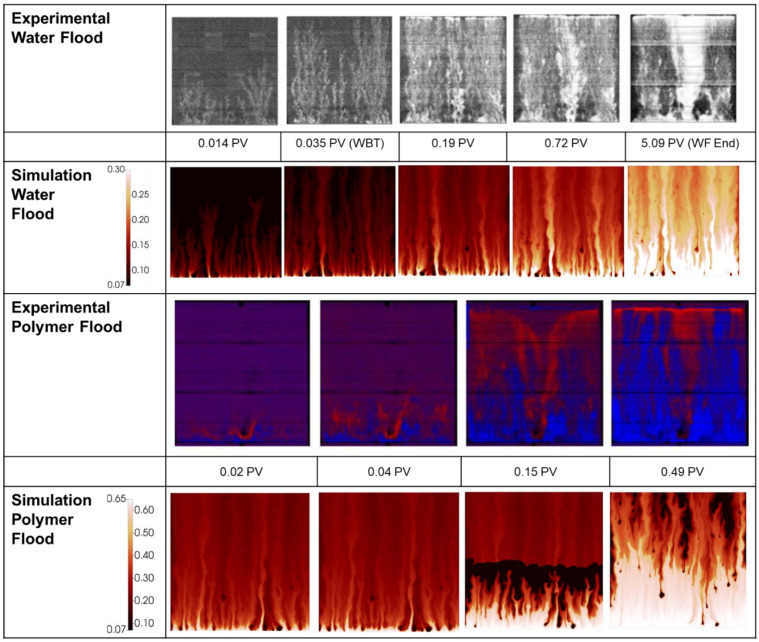
Experimental vs. simulated finger patterns during displacement of 7000 mPa.s oil.

Figure 8 compares the simulated finger patterns against the X-ray saturations observed in the experiment. As with the 2000 mPa.s case, the polymer flood images have been generated via subtraction of the end of water food image.

**Figure 9:**
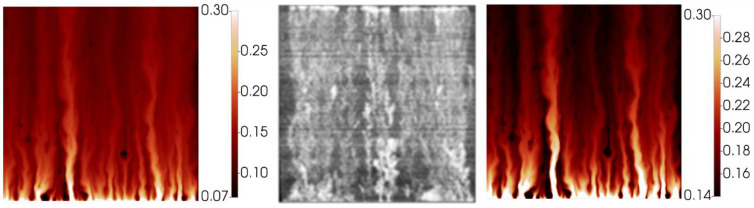
Demonstration of the impact of the low water saturation threshold on the visualised finger patterns.

As with the 2000 mPa.s case, the early portion of the water flood for the 7000 mPa.s oil shows representative finger patterns when compared to the experimental images. As the flood develops, a more disperse finger pattern is obtained in the simulation vs. the experiment, however as noted above, this may be in part due to the image threshold/sensitivity to the very lowest water saturations. Figure 9 demonstrates the significant change in observed finger pattern with a change in threshold of just 0.07 saturation units.

During the injection of polymer viscosified water, Figure 8, the distribution of fingers, oil bank and instability of the polymer bank is well captured at all stages of the polymer flood. Some delay in the formation of the oil bank vs. the experimental images is observed despite the good agreement observed between the simulation and the effluent samples, Figure 7.

**Figure 10:**
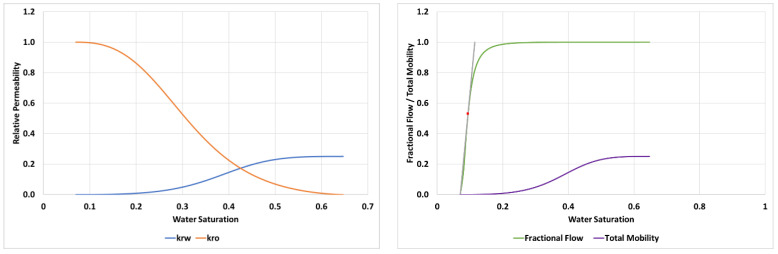
Relative permeabilities (**left**) and fractional flow & total mobility (**right**) for the displacement of 7000 mPa.s oil.

LET parameters used to perform this simulation are given in Table 4 and the resultant relative permeabilities, fractional flow and total mobility functions are shown in Figure 10.

In the case of 7000 mPa.s oil, the fractional flow gives $S_{wf}\;$= 0.094 and a corresponding value of $M\left(S_{wf}\right)$ = 1.14.

### 3.4. Displacement of µ_o_ = 412 and 616 mPa.s Oils

Upon examination of the experimental finger patterns observed in the cases of 412 and 616 mPa.s oils, it was clear that some larger scale heterogeneity features must be present, since the flows clearly appeared to “avoid” certain regions of the slab, and “prefer” others. As a result, it was only possible to obtain similar finger patterns in the simulations when some degree of structured heterogeneity was included in the model superimposed upon the random correlated permeability field. As shown above, this was not necessary for the 2000 and 7000 mPa.s simulations, which only required a single random correlated permeability field.

**Figure 11:**
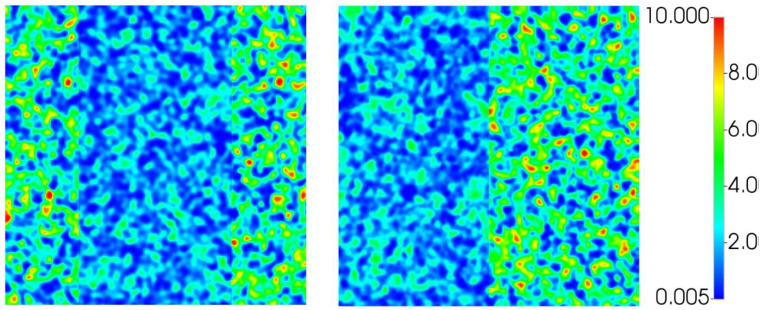
Structured permeability fields (D) used in simulation of the 412 mPa.s (**left**) and 616 mPa.s (**right**) cases.

The observed flows for the water injection stages of the 2D slab floods directly suggested the simple changes to the permeability field required. The resulting modified permeability fields are shown in Figure 11. The same base permeability field is used, as in Figure 1, however in the 412 mPa.s case the center 50% of the grid was reduced in permeability by half. While the permeability in the 616 mPa.s case is reduced by half on the left 50% of the grid. Recall that the 412 mPa.s experiment was performed in a smaller slab (15 cm × 15 cm) than the other 3 experiments (30 cm × 30 cm), and therefore a coarser grid was used (250 × 250 cells) to maintain the same grid dimensions across the different simulations. We originally regarded making these changes as being somewhat arbitrary and rather unsatisfactory, and considered omitting them from this paper. However, the water and tertiary polymer displacement simulation results are reported here for two reasons, (i) they match the experiments extremely well in all respects, as shown below, and more importantly, (ii) they actually demonstrate the presence of a “double” viscous crossflow (VX) mechanism which leads to a large response to polymer for each of these 2 cases (explained below).

**Figure 12:**
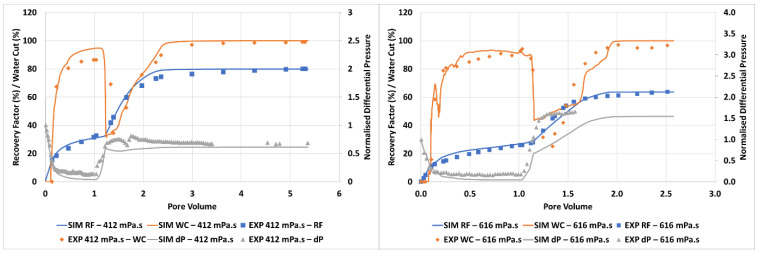
Simulation match of the unstable displacement of 412 mPa.s oil (**left**) and 616 mPa.s oil (**right**).

The simulation matches to the experiment using the grid modifications described above are shown in Figure 12. The oil recovery number in Figure 12 (summarised in Table 2) indicate incremental recoveries due to the tertiary floods (IR) of ~100% in each case. As we noted above, this is an extremely high value of IR for any EOR process. However, as for the other higher oil viscosity cases, the simulations using the modified permeability model gave exactly the same quantitative recovery responses. However, as we explain below, there is additional mechanism at work in these cases due to the assumed (and observed) permeability heterogeneity.

**Figure 13:**
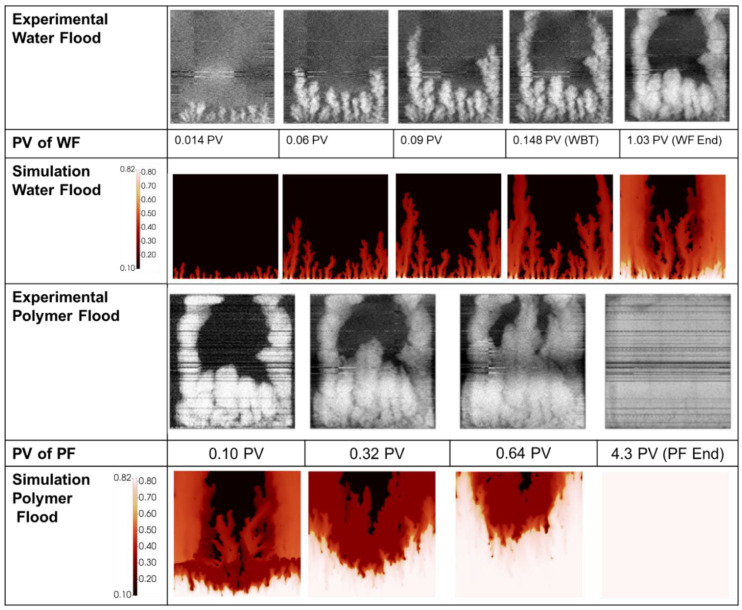
Experimental vs. simulated finger patterns during displacement of 412 mPa.s oil.

**Figure 14:**
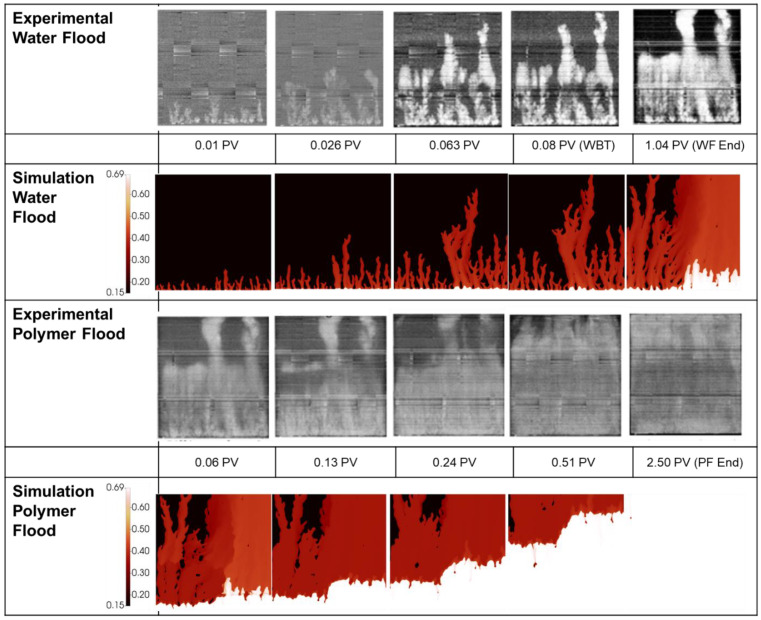
Experimental vs. simulated finger patterns during displacement of 616 mPa.s oil.

It can be seen that a very good (but not perfect) match between experiment and simulation is obtained for both floods for the oil recovery, water cut, and pressure profiles. The corresponding finger patterns observed in the simulation vs. experiment are presented in Figure 13 for the 412 mPa.s case and Figure 14 for the 612 mPa.s case. By studying the animations of the evolution of the finger patterns, the water/oil saturations, and the oil banking, it became clear what mechanisms led to the very high IR values for the polymer response described above (IR~100%). The animations of water saturation against time are supplied as Supplementary Materials with this paper and may be downloaded from the journal website (Videos S1–S4).

*Analysis of the 412 mPa*.*s case*: The finger patterns during water flooding of the 412 mPa.s oil is presented in Figure 13 show excellent agreement with the experiment with some acceleration in propagation of the central fingers vs. the edge fingers at the end of the water flood. As in the previous simulation cases, the finger pattern at the end of the water flood is slightly more dispersed for the dominant fingers than in the simulation. However, even well after water breakthrough (at 1.03 PV), very little injected water has entered the low permeability central channel. This is because the higher permeability channels on either side are full of low viscosity (1 mPa.s) water and the bypassed lower permeability central channel contains much more viscous (412 mPa.s) oil. It is this situation which maximises bypassing and reduces any possibility of additional water injection displacing the oil in this central region by continued water injection. However, this situation does increase the target oil for the subsequent polymer injection, as discussed below.

As the viscosified polymer flood begins, the results in Figure 13 show two important effects in *both* the experiments and the simulations, as follows: (i) the finger patterns change and viscous fingering is now observed in the central low permeability channel, and (ii) very clear oil banking occurs and some of this is due to larger scale crossflow of oil from the central low permeability region into the swept regions on either side of the system. As with the experiment, at the end of the flood the entire slab is swept to an almost constant water saturation. The reason for the appearance of fingering in the central region is that the polymer banks the already injected water and the connate water, and it is this which causes the fingers in this central region of the system. The simulations reproduce all of this behaviour extremely well, and it is our observations from the corresponding animations that reveal that ***two*** viscous crossflow (VX) mechanism are at work here at different scales in this system, viz. (1) firstly the polymer mechanism at the scale of the individual fingers is causing banking of oil by a local VX mechanism (as it did in the 2000 and 7000 mPa.s cases above); and (ii) there is an *additional* “layer to layer” VX mechanism causing displacement of the oil in the low permeability central zone. This latter “layer to layer” VX mechanism is the one that has been well described in the literature for many years (Sorbie, 1991 [23] and many references therein). Superimposed on the VX mechanism is, of course, the direct displacement “extended Buckley-Leverett” mechanism as described above in the introduction, but this is the weaker mechanism in terms of its contribution to IR. In summary, it is this “double VX” mechanism due to the viscous polymer that is additionally contributing to this very large IR response.

It is fortuitous that this occurs in this 2D slab flood, since it has brought to light an experimental case showing the “double VX” mechanisms which has helped us to explain the response to polymer. However, it also implies that we must take some care in comparing the results for this case (and the 616 mPa.s case below) directly with the 2000 mPa.s and 7000 mPa.s results, because of the additional factor in these cases, i.e., the heterogeneity in the 2D slab systems.

*Analysis of the 616 mPa*.*s case*: During water flooding, the simulated finger patterns for the 616 mPa.s case presented in Figure 14 show good agreement with those obtained experimentally by in situ imaging of the 2D slab floods. In this case, the permeability field has a higher permeability channel on the right half of the system, as described above. In this high permeability we observe one larger, splitting finger in the simulations compared with the two more stable fingers observed in the experiment. This is a relatively minor deviation that is simply due to the specific random permeability field used in this set of experiments. However, both the simulation and experiment show clear preferential bypassing on the right of the model leaving most of the bypassed oil on the left of the model. As with the 3 previous displacement cases, by the end of the water flood the primary fingers have become more dispersed than those presented in the experimental images, however, as mentioned above this could be due to the processing of the experimental images.

During polymer viscosified water injection, crossflow from the low permeability zone (left) to the high permeability zone (right) can be seen at 0.06 PV and by 0.13 PV (see Figure 14) which has resulted in an even oil saturation across all flooded areas. Following this, the slab is systematically swept in line with the experimental measurements. Again, like the 412 mPa.s oil displacement, the response of the 616 mPa.s oil recovery to polymer can be analysed in terms of the “double” viscous crossflow (VX) mechanism as descried above.

**Figure 15:**
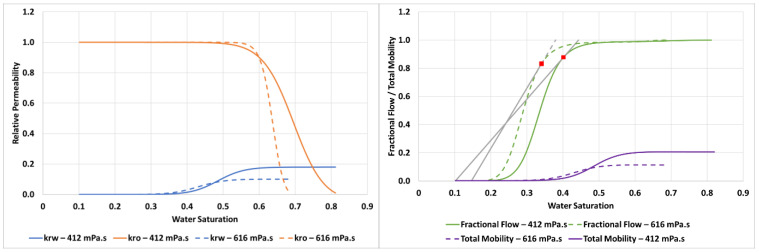
Relative permeabilities (**left**) and fractional flow & total mobility (**right**) for the displacement of 412 mPa.s and 616 mPa.s oil.

The LET parameters for both floods (µ_o_ = 412 mPa.s and 616 mPa.s) are shown in Table 5 and the resulting relative permeabilities, fractional flows and total mobilities are shown in Figure 15.

From the fractional flow tangent, it can be calculated that the $S_{wf}$ is 0.40 and 0.35 for the 412 mPa.s and 616 mPa.s cases, respectively, while the $M\left(S_{wf}\right)$ is 6.36 and 4.97, respectively.

## 4. Discussion

**Figure 16:**
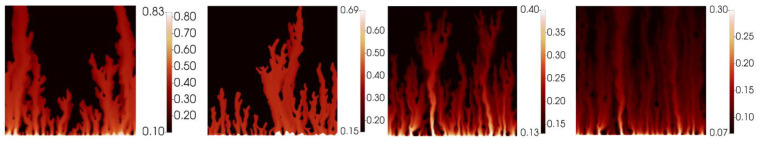
Finger patterns for displacement of 412 mPa.s, 616 mPa.s, 2000 mPa.s and 7000 mPa.s oil (**left** to **right**) at the point of water breakthrough (0.148, 0.08, 0.043, & 0.035 PV, respectively).

**Figure 17:**
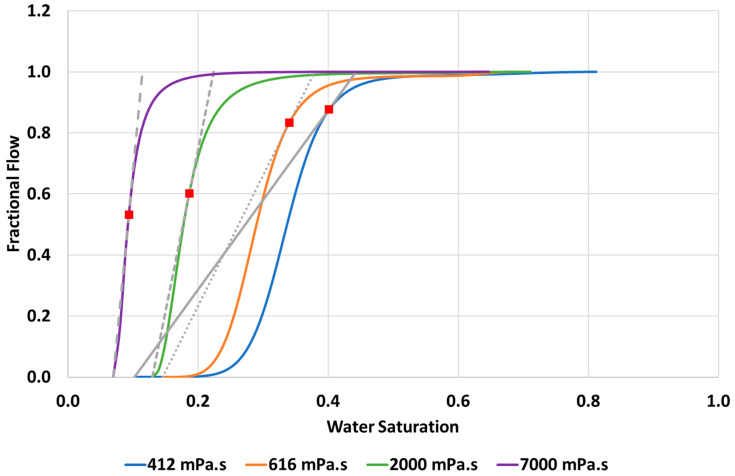
Summary of the matched fractional flow curves (f_w_*) for the 4 water flooding cases simulated in this work.

*The 4 Waterfloods*: The simulation approach described in this paper has been used to match the immiscible fingering data observed in all 4 waterfloods over a wide range of viscosity ratios. The model has reproduced all of the experimental data including the profiles of oil recoveries, water cuts and pressure drops (vs. PV) while showing realistic and representative finger patterns during the water flooding stage of the displacement process. It can be seen from the simulations, summarised from above in Figure 16, that as the viscosity ratio between displaced and displacing fluid increases (µ_o_/µ_w_ from 474 to 7000) the finger patterns become thinner, wispier, and more numerous, as is the case in the experimental observations. In other words, the water saturations in the actual fingers get lower as viscosity ration increases as is shown in the trend of the matched fractional flows, f_w_*, for the 4 cases simulated in Figure 17 and in the numerical results in Table 6. The values of the frontal shock water saturation in the fingers for each case, S_wf_, are also shown in this table along with the *local* shock front mobility ratio M(S_wf_) defined in Equation (4) and also the “conventional mobility ratio”, M, defined using the end point saturations of the relative permeability curves, i.e., $M=\left(\frac{\lambda_{w}\left(S_{w1}=1-S_{or}\right)}{\lambda_{o}\left(S_{w2}=S_{wi}\right)}\right)=\left(\frac{k_{rw}\left(S_{w1}\right).\;\mu_{o}}{k_{ro}\left(S_{w2}\right).\;\mu_{w}}\right)$.

As shown here, and discussed previously in Salmo et al. (2022) [15], going from the lowest to highest oil viscosity, then clearly the viscosity ratio, (µ_o_ /µ_w_), and the (conventional end-point) mobility ratio, ***M***, both increase. This is actually anti-correlated with the value of the local mobility ratio M(S_wf_) at the shock front, where S_w_ = S_wf_ (see Table 1). Our results also show that the magnitude of ***M*** is ***mostly*** related to the viscosity ratio itself, and it is also evident that there is no local point in the system where ***M*** actually occurs since it refers to 2 different S_w_ values (although the quantity M(S_wf_) or any M(S_w_) are truly *local*). We therefore favour referring to the immiscible instability being due to viscosity ratio (µ_o_ /µ_w_) rather than (conventionally defined) mobility ratio ***M***. The shock front mobility ratio, M(S_wf_), would be a suitable quantity to refer to but, as we note, this quantity actually *decreases* as the viscosity ratio (or ***M***) increases, which may suggest that the front becomes *more stable*. This was previously referred to in our earlies work as the “M-paradox”, but it is essentially a confusion of nomenclature [16].

**Figure 18:**
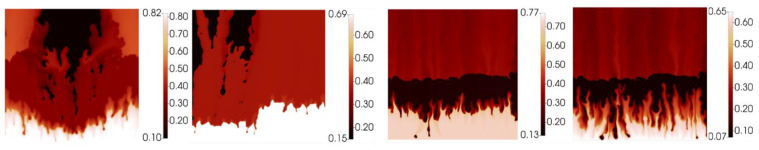
Finger patterns for displacement of 412 mPa.s, 616 mPa.s, 2000 mPa.s and 7000 mPa.s oil (**left** to **right**) at 0.15 PV of polymer viscosified brine injected.

*The 4 Tertiary Polymer Floods*: We note that, after successfully matching the 4 waterfloods, the subsequent behaviour of the tertiary polymer injection is essentially a **prediction**; the only additional data used was the polymer viscosity, the (low) adsorption level and the final RRF (residual resistance factor [23]). The simulation predictions correctly reproduced the experimental rapid increase in oil recovery observed in the experiments, the corresponding drop in water cut, the pressures (all vs. PV) as well as the adjusted finger pattern caused by the viscous polymer. In particular, the early formation of an oil bank is well reproduced in the simulation in line with in situ images as shown in Figure 18 where the oil bank is seen at 0.15 PV after polymer injection.

Through comparison of the visualised simulations in Figure 18 it is clear that the oil bank formation is strongly dependent on the viscosity of the displaced fluid. In the 2000 mPa.s and 7000 mPa.s cases it can be observed that a strong oil bank is formed at approximately the same rate of propagation. However, it is also seen that the front of the polymer viscosified water bank is still highly unstable, with fingers propagating a significant way into the oil bank. For the lower two oil viscosities, the situation is slightly different because of the larger scale slab heterogeneity, and this is discussed below.

*Tertiary Polymer Floods for the* µ*_o_ = 412 mPa*.*s and 616 mPa*.*s Cases*: In order to achieve more exact finger patterns for these 2 lower viscosity oil cases, it was necessary to modify the permeability field to encourage finger growth in these specific locations. Without direct access to the original slabs, it is difficult to ascertain whether this distribution is indeed due to permeability, or some other factor—e.g., porosity variance, saturation distribution or experimental artifact. Nevertheless, all aspects of these 2 experiments have been very satisfactorily captured for both the waterflood and the tertiary polymer floods. An additional feature of interest is that both of these cases demonstrate the viscous crossflow (VX) mechanism at *two* length scales; viz. at the scale of the individual finger and also at the layer scale, and this is referred to as a “double VX” mechanism.

## 5. Conclusions

The specific conclusions from this study are summarised, as follows:

1. It has been demonstrated that the methodology proposed by Sorbie et al. (2020) [14] can be utilised to generate very close matches to most measured aspects of a series of unstable displacement 2D slab floods (µ_o_/µ_w_ ~470 to 7000), namely oil production, water cut, pressure drop and finger patterns in both water flooding and tertiary polymer flooding.
2. All of the experimental observations have been honoured, i.e., profiles of oil recovery, water cut, pressure drop and finger patterns (vs. PV), for the water floods. However, the subsequent behaviour of the tertiary polymer floods are essentially ***predictions*** which correctly match the incremental oil response, the drops in water cut and the oil bank formation.
3. The results in this paper tend to support our view that the immiscible fingering is best characterised by the viscosity ratio (µ_o_/µ_w_) rather than by the “conventional mobility ratio” (***M***), but both are reported here for completeness.
4. Analysing the animations of the tertiary polymer floods strongly support the view that the polymer works by 2 main mechanisms, viz. (a) enhanced viscous linear displacement (or extended Buckley-Leverett (BL) mechanism; see [22,41]; and (b) by an additional viscous crossflow (VX) mechanism. The second mechanism contributes much more to the large incremental recoveries (IR) in these viscous oil floods, which show IR values >100%. Such improvements in IR are not possible based purely on the extended BL mechanism.
5. In two of the floods (the µ_o_ = 412 mPa.s and 616 mPa.s cases) we had to invoke some higher level of heterogeneity (essentially some degree of superimposed permeability layering) to accurately reproduce the waterflood behaviour. Very good matches to these two waterfloods were then achieved and excellent predictions of the tertiary polymer floods were found. These 2 tertiary polymer floods showed a “double VX” mechanism in producing the incremental oil, comprising of a VX effect at the scale of the single fingers and an additional VX mechanism at the scale of the permeability layer. This latter layer to layer mechanism is indeed the “conventional” VX mechanism reported widely for many years [23]. However, this “double VX” mechanism emerged quite straightforwardly in the analysis of these experiments and is reported here in (literature) experiments and theoretically for the first time.

## Figures and Tables

**Table 1:** Oil, water, and polymer solution viscosities.

Oil Viscosity	Water Viscosity	Viscosity Ratio	Polymer Solution Viscosity
µ_o_	µ_w_	µ_o_/µ_w_	µ_p_
mPa.s	mPa.s		mPa.s
412	0.88	468	18
616	0.88	700	28
2000	1.02	~2000	60
7000	1.02	~7000	60

**Table 2:** Summary of experimental results.

µ_o_	µ_w_	µ_p_	µ_o_/µ_w_	µ_o_/µ_p_	EW	PR	(PR-EW)	100 × (PR-EW)/EW
mPa.s	mPa.s	mPa.s			%	%	%	%
412	0.88	18	468	23	40	80	40	100
616	0.88	28	700	22	31	63	32	103
2000	1.02	60	1961	33	29	66	37	128
7000	1.02	60	6863	117	26	61	35	135

**Table 3:** LET input parameters for the 2000 mPa.s oil displacement case.

2000 mPa.s Case									
S_wi_	S_or_	k_ro_*	k_rw_*	L^w^_o_	E^w^_o_	T^w^_o_	L^o^_w_	E^o^_w_	T^o^_w_
0.13	0.289	1	0.14	1	2	14.5	2.2	1.5	3

**Table 4:** LET input parameters for the 7000 mPa.s oil displacement case.

7000 mPa.s Case									
S_wi_	S_or_	k_ro_*	k_rw_*	L^w^_o_	E^w^_o_	T^w^_o_	L^o^_w_	E^o^_w_	T^o^_w_
0.07	0.353	1	0.25	1.4	4	2.4	2	3	3

**Table 5:** LET input parameters for the 412 mPa.s and 616 mPa.s oil displacement case.

Oil Viscosity	S_wi_	S_or_	k_ro_*	k_rw_*	L^w^_o_	E^w^_o_	T^w^_o_	L^o^_w_	E^o^_w_	T^o^_w_
616 mPa.s	0.146	0.31	1	0.1	1	1	22	4	1	3.5
412 mPa.s	0.102	0.18	1	0.18	1	1.3	10	6	0.5	4

**Table 6:** Water saturations, finger mobilities and end point mobility (***M***) for the 4 water flooding cases.

Oil Viscosity (mPa.s)	$\frac{{µ}_{o}}{\mu_{w}}$	$S_{wf}$	$M\left(S_{wf}\right)$	M
412	474	0.40	6.36	65.3
616	700	0.35	4.97	54.2
2000	2000	0.19	1.50	280
7000	7000	0.094	1.14	1750

## Supplementary Materials

The following supporting information can be downloaded at: https://www.mdpi.com/article/10.3390/polym14194159/s1. Video S1: 412 mPa.s Oil Displacement, Video S2: 616 mPa.s Oil Displacement, Video S3: 2000 mPa.s Oil Displacement, Video S4: 7000 mPa.s Oil Displacement.
